# Effect of silibinin and *trans-*chalcone in an Alzheimer's disease-like model generated by insulin amyloids

**DOI:** 10.1590/1414-431X2023e12443

**Published:** 2023-02-27

**Authors:** M. Omidi-Shahsavandi, P. Yaghmaei, S. Ahmadian, A. Ebrahim-Habibi

**Affiliations:** 1 Department of Biology, Faculty of Basic Sciences, Science and Research Branch, Islamic Azad University, Tehran, Iran; 2 Institute of Biochemistry and Biophysics, University of Tehran, Tehran, Iran; 3 Biosensor Research Center, Endocrinology and Metabolism Molecular-Cellular Sciences Institute, Tehran University of Medical Sciences, Tehran, Iran; 4 Endocrinology and Metabolism Research Center, Endocrinology and Metabolism Clinical Sciences Institute, Tehran University of Medical Sciences, Tehran, Iran

**Keywords:** Alzheimer's disease, Amyloid, Fibril inhibitor, Hippocampus, Insulin, Silibinin

## Abstract

Amyloid fibrils are characteristic of several disorders including Alzheimer's disease (AD), with no cure or preventive therapy. Diminishing amyloid deposits using aromatic compounds is an interesting approach toward AD treatment. The present study examined the anti-fibrillogenic effects of silibinin and *trans-*chalcone *in vitro*, *in vivo*, and *in silico* on insulin amyloids. *In vitro* incubation of insulin at 37°C for 24 h induced amyloid formation. Addition of *trans-*chalcone and silibinin to insulin led to reduced amounts of fibrils as shown by thioflavin S fluorescence and Congo red absorption spectroscopy, with a better effect observed for silibinin. *In vivo* bilateral injection of fibrils formed by incubation of insulin in the presence or absence of silibinin and *trans-*chalcone or insulin fibrils plus the compounds in rats' hippocampus was performed to obtain AD characteristics. Passive avoidance (PA) test showed that treatment with both compounds efficiently increased latency compared with the model group. Histological investigation of the hippocampus in the cornu ammonis (CA1) and dentate gyrus (DG) regions of the rat's brain stained with hematoxylin-eosin and thioflavin S showed an inhibitory effect on amyloid aggregation and markedly reduced amyloid plaques. *In silico*, a docking experiment on native and fibrillar forms of insulin provided an insight onto the possible binding site of the compounds. In conclusion, these small aromatic compounds are suggested to have a protective effect on AD.

## Introduction

Incorrect folding and protein aggregation are directly related to a large number of degenerative diseases of the nervous system such as Alzheimer's, Parkinson's, and Huntington's diseases as well as peripheral diseases such as systemic amyloidosis and type 2 diabetes. Alzheimer's disease (AD) is the major cause of dementia and is associated with progressive impairment of intelligence, memory, and thinking skills (1).

The incorrect folding and aggregation of various sorts of proteins (and peptides) occurs in the form of filamentous structures called amyloid fibrils. Studies in recent years have reported that the instability of the natural spatial structure of proteins, which leads to their aggregation, is a very important factor in functional disorders related to protein aggregation (1). Amyloid-forming proteins are not limited to disease-related proteins. Many other proteins have the potential to create amyloid aggregates under denaturation conditions *in vitro* and insulin is one of them. In laboratory studies, it has been demonstrated that under denaturation conditions such as acidic pH and high temperature, insulin monomeric molecules accumulate to form amyloid fibrils (2).

Inhibition of this process is considered a potential therapeutic solution in the treatment of amyloid-related diseases. The most important strategies to inhibit protein aggregation and amyloid fibril formation are to stabilize the natural folding state of proteins, act on the monomer protein population, inhibit the formation of amyloid fibrils, and finally direct amyloid species to pathways causing the formation of amorphous and non-toxic protein aggregates (3). Thus, various studies have been performed to identify compounds that inhibit amyloid structures, particularly on proteins linked to degenerative disorders of the nervous system (4). Several small natural molecules, especially natural polyphenols, have been reported to inhibit protein aggregation (1), since herbal medicine has received extensive focus compared with chemical drugs because of their fewer side effects (5). In the current study, two aromatic natural compounds were studied in a model of AD generated by insulin amyloid fibrils.

One such compound is the *trans-*chalcone (1,3-diphenyl-2-propen-1-one), the biphenolic core structure of flavonoid precursors. Studies on *trans-*chalcone have shown different properties such as antioxidant and acetylcholinesterase inhibitory effects as well as anti-amyloid effects on amyloid-β (Aβ) formation for this compound (6). The second compound is silibinin, a natural polyphenolic flavonoid isolated from seed extracts of the herb milk thistle (*Silybum marianum*), which is the major active constituent of silymarin. The curative effects of silymarin have been known for over two thousand years (7).

Silibinin has many therapeutic properties, the most prominent of which is the treatment of liver disorders. Due to its antioxidant properties, this compound is also effective in reducing oxidative stress in liver cells, macrophages, and neuronal cells. In recent years, silibinin has been shown to be effective in treating various types of cancer (8). Regarding degenerative diseases of the nervous system, Urata et al. (9) reported that silymarin could inhibit the aggregation of amyloid beta peptide and reduce the toxicity of the resulting protein aggregates on PC12 cells.

In the present study, similar to the usual experiments involving Aβ peptide, insulin fibrils were first produced *in vitro* and then injected bilaterally into Wistar rats' hippocampus in order to obtain a rat model with Alzheimer's-like symptoms. Subsequent to injection, memory impairment and plaque formation in the brain tissue were studied via shuttle box test and amyloid plaque staining, respectively. Fibrils formed in the presence of *trans-*chalcone and silibinin were then tested in the model in order to assess the potential of these compounds as generic anti-amyloids.

## Material and Methods

### Chemicals

Silibinin, *trans-*chalcone (benzylideneacetophenone), thioflavin S, and Congo red were purchased from Sigma (USA). Regular insulin was purchased from EXIR Pharmaceutical Company (Iran). Chloroform, monopotassium phosphate (KH_2_PO_4_), potassium phosphate (KH_2_PO), and dimethyl sulfoxide (DMSO) were purchased from Merck (Germany).

### *In vitro* methods (insulin amyloid formation)

Initially, insulin solution was prepared with a final concentration of 0.5 mg/mL in phosphate buffer (50 mM, pH 7.4) in the presence or absence of 1 mM concentration of silibinin and *trans-*chalcone. The vials containing the insulin samples were then incubated for 24 h at 37°C on a stirrer rotating at 100 rpm (10). Since the Congo red marker binds specifically to amyloid fibrils, resulting in an increase in λ_m_ as well as in absorbance levels, Congo red absorption rate of incubated protein samples was assayed in the absence and presence of silibinin and *trans-*chalcone (1 mM). To do so, 500 μL of Congo red solution (20 μM in 5 mM potassium phosphate and 150 mM sodium chloride, pH 7.4) plus 25 μL of the sample was poured into a 0.5 mL microtube and filtered using a 2-μM pore syringe filter, then stirred and placed in the laboratory for 10 min until color stabilization. The Congo red absorption spectrum was then recorded at 600-400 nm using the Shimadzu UV-1800 spectrophotometer (11).

### Experimental groups

In this study, adult male Wistar rats weighing 250-300 g were purchased from the Pasteur Institute of Iran. They were kept under suitable environmental conditions with a temperature of 22±2°C, 50% humidity, and a 12-h light/dark cycle with free access to water and food. After a one-week adaptation period, the rats were randomly divided into 5 groups of six rats each (control, model, EXP1, EXP2, and EXP3) (Table 1).

**Table 1:** Description of the experimental groups with Wistar rats.

Groups	Definition
Control	Healthy rats
Model	Injection of 4 μL of 24-h incubated insulin into hippocampus
EXP1	Silibinin and *trans*-chalcone (1 mM) added to insulin and incubated for 24 h, then injection of 4 μL of this solution into the hippocampus
EXP2	Silibinin and *trans*-chalcone added to 12-h incubated insulin then continued incubation for 12 h, followed by injection of 4 μL of this solution into the hippocampus
EXP3	Silibinin and *trans*-chalcone added at the moment of injection to 24-h incubated insulin followed by injection of 4 μL of this solution into the hippocampus

For stereotactic surgery, the rats were first weighed and then anesthetized by intraperitoneal injection of ketamine (100 mg/kg) and xylazine (20 mg/kg). The animal was fixed to the device and then a longitudinal incision was made in the posterior part of the skull, according to the Paxinos and Watson Rat Brain Atlas (AP=-4.2, ML=3, DV=3.5) (12). A mixture of insulin fibril and *trans-*chalcone was injected according to Table 1 with a Hamilton syringe. The injection rate was 0.5 μg/min (13). At the end of the third week after surgery, all groups underwent behavioral tests related to learning and memory.

The experimental protocol was approved by the Animal Ethics Committee of the Science and Research Branch at the Islamic Azad University (Tehran, Iran). The research was performed according to Guidelines of the National Institutes of Health on the principles of laboratory animal care (NIH Publication No. 80-23, revised in 1980).

### Avoidance learning behavioral test

Passive avoidance memory was evaluated with the Shuttle box instrument. This test was performed on two consecutive days.

Adaptation session: on the 21st day after the injection of fibrils or fibrils plus compounds (Table 1), while the space between the light and dark chambers was open, the rats were placed in the light chamber and allowed to stay for two minutes to adapt to the device and find the opening between the dark and light chambers. Then, the rat was taken out of the device and transferred to a solitary cage. Training session: 30 min after the adaptation period, each rat was placed in a light chamber while the guillotine door was closed. Ten seconds later, the guillotine door was opened; once the rat went into the dark chamber, the door was closed immediately. An electric shock was applied for 2 s with a current of 1 mA at a frequency of 50 Hz from the metal rods on the sole of the rat's feet, and 20 s later the rat was taken out of the dark chamber. To assess if the rat had learned that it should not enter the dark chamber, two minutes after removing the rat from the dark chamber, the rat was placed again in the light chamber and given two minutes to see if it would enter the dark chamber or if it had learned from the initial shock that it should not enter it. If the rat entered the dark chamber a second time after the shock, the guillotine door was closed again, and the shock was applied again. Memory retention session: 24 h after the training phase, each rat was placed in the light chamber (while the guillotine door was closed) and 10 s later, the guillotine door was opened and the delay time for entering the dark chamber was recorded as “step-through latency time”. The maximum time delay for entering the dark chamber was 300 s (14).

### Histological studies

The brain was removed for histological examination and placed in fixative formalin for 24 h. Next, during the dehydration and clarification steps, the samples were embedded into molten paraffin. Using a microtome (Cambridge Medical Instruments, United Kingdom), 6-micron-thick sections were prepared. The samples were placed on a slide, and then half of the samples were stained with hematoxylin-eosin and the other half with thioflavin S.

The main goal of preparing sections of the hippocampus and staining them was to investigate possible changes in brain tissue and to quantitatively examine the amyloid fibrils formed in the absence and presence of silibinin and *trans-*chalcone in each section. Five tissue sections in each group were randomly examined microscopically. Since we observed that silibinin performed better than *trans-*chalcone in the initial tests, both DG and CA1 regions were checked for the former and DG for the latter.

### Docking

**Figure 1:**
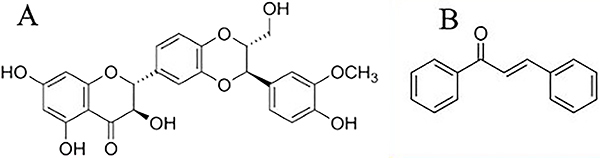
2D structure of (**A**) silibinin and (**B**) *trans-*chalcone.

Molecular docking was performed using the Autodock Vina software (USA). The molecular structure of silibinin and *trans-*chalcone was prepared from pubchem (https://pubchem.ncbi.nlm.nih.gov/) (Figure 1). Then, energy minimization was performed using Avogadro software (https://avogadro.cc) to provide a stable 3D structure. For insulin, two natural (3I40) and denatured (1SF1) structures were fetched from the RCSB protein database (https://www.rcsb.org). First, water molecules in the crystal structure were removed manually. Then, polar hydrogens and Gasteiger charges were added for both ligands and proteins using Autodock Tools software. All rotating bands of the active ligand were considered. Docking was performed blindly, and the search space was considered large enough for the entire protein, as all the protein surface was available to the ligand. The 2D protein-ligand interactions were demonstrated using LIGPLOT software (https://bio.tools). 3D images were also drawn using VMD software (https://www.ks.uiuc.edu).

### Statistical analysis

Data were statistically analyzed by SPSS software (IBM, USA) using one-way analysis of variance (ANOVA), followed by *post hoc* Tukey test. The results are reported as means±SE. P<0.05 was considered significant. The diagrams were drawn using GraphPad Prism 5 software (USA).

## Results

### *In vitro* amyloid formation by Congo red and thioflavin S testing

**Figure 2:**
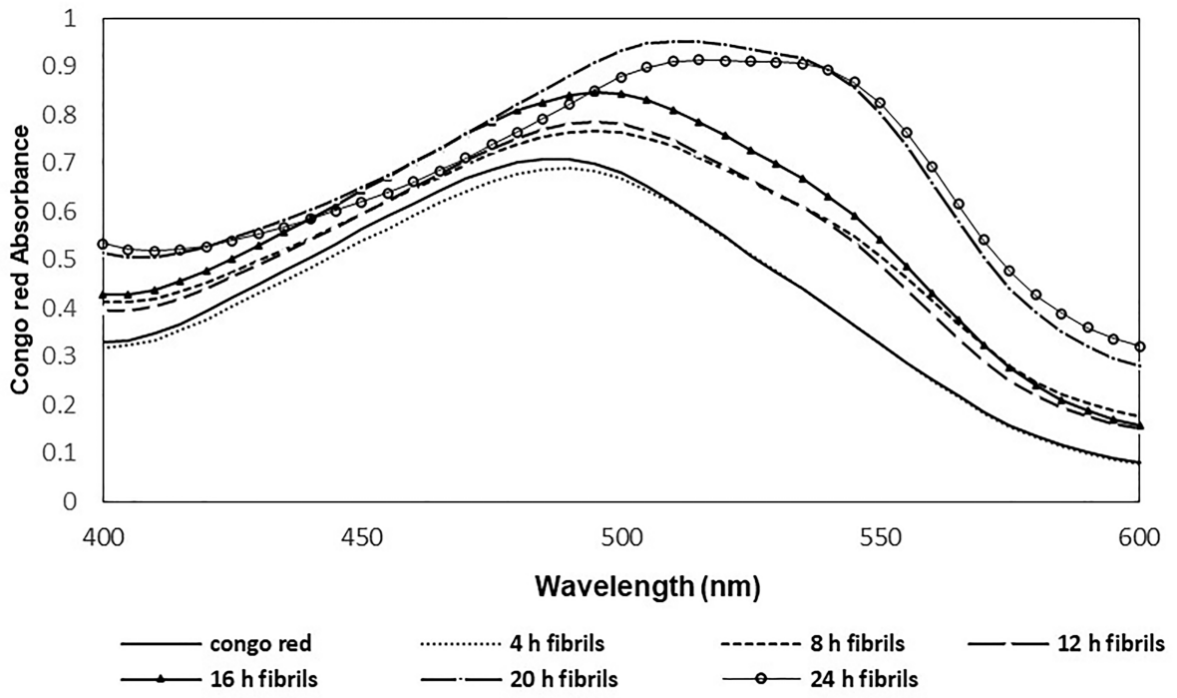
Congo red absorption spectrum of incubated insulin at 4, 8, 12, 16, 20, and 24 h.

**Figure 3:**
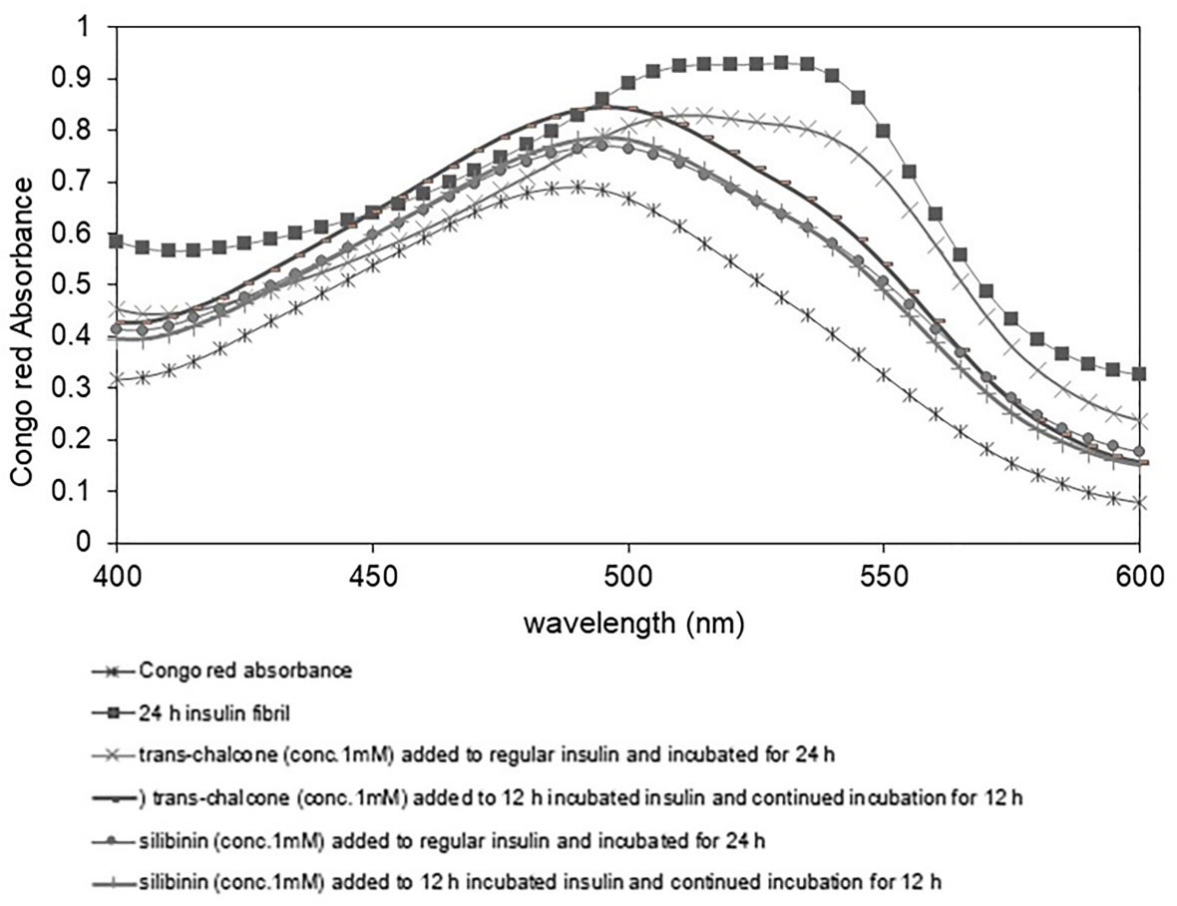
Congo red binding adsorption spectra of regular insulin in the absence and presence of *trans-*chalcone and silibinin.

Significant and gradual increase in Congo red absorbance associated with a red shift was detected in the amyloidogenic conditions over time (from 4 to 24 h), indicating the presence of a considerable amount of amyloid fibrils formed as a result of insulin incubation (Figure 2). However, in the presence of *trans-*chalcone and silibinin, decreased absorbance and shift was observed, while adding *trans-*chalcone and silibinin from the beginning of the process to the medium showed a better inhibition of fibril formation. The lower absorbance observed after addition of the compound at mid-process (compared with no additives) is also indicative of the inhibitory potential of *trans-*chalcone and silibinin in the very formation of amyloid structures (Figure 3).

During the experiment, various concentrations of *trans-*chalcone and silibinin were added to the incubation medium during fibrillation (results not shown), and the 1 mM concentration was found to be effective in attenuating insulin fibril formation, with silibinin showing a superior effect compared with *trans-*chalcone. Further studies were thus performed with this concentration.

**Figure 4:**
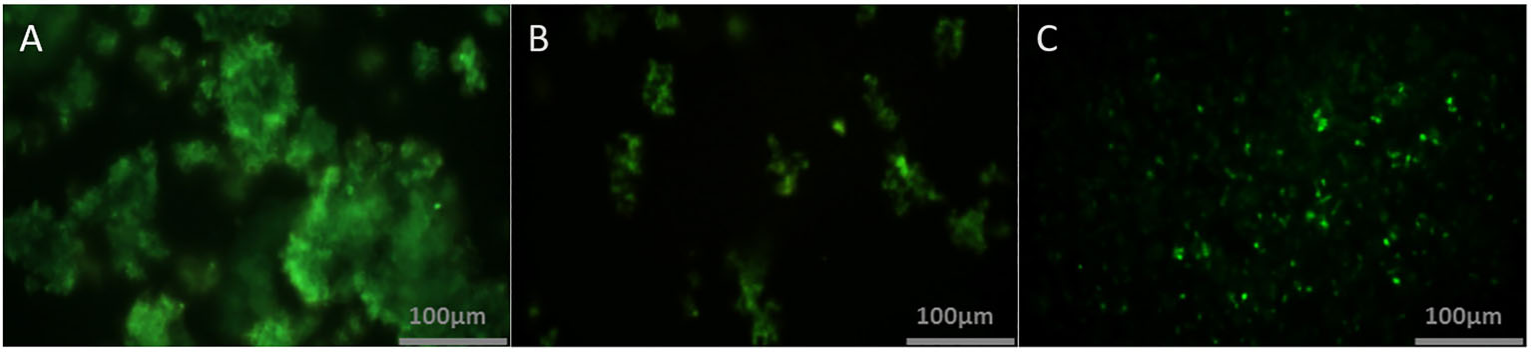
Fluorescence microscopy results, revealing amyloid fibril deposits by thioflavin S staining. **A**, insulin amyloid images; **B,** insulin amyloid images in the presence of *trans-*chalcone; **C**, insulin amyloid images in the presence of silibinin. Scale bar 100 μm.

**Figure 5:**
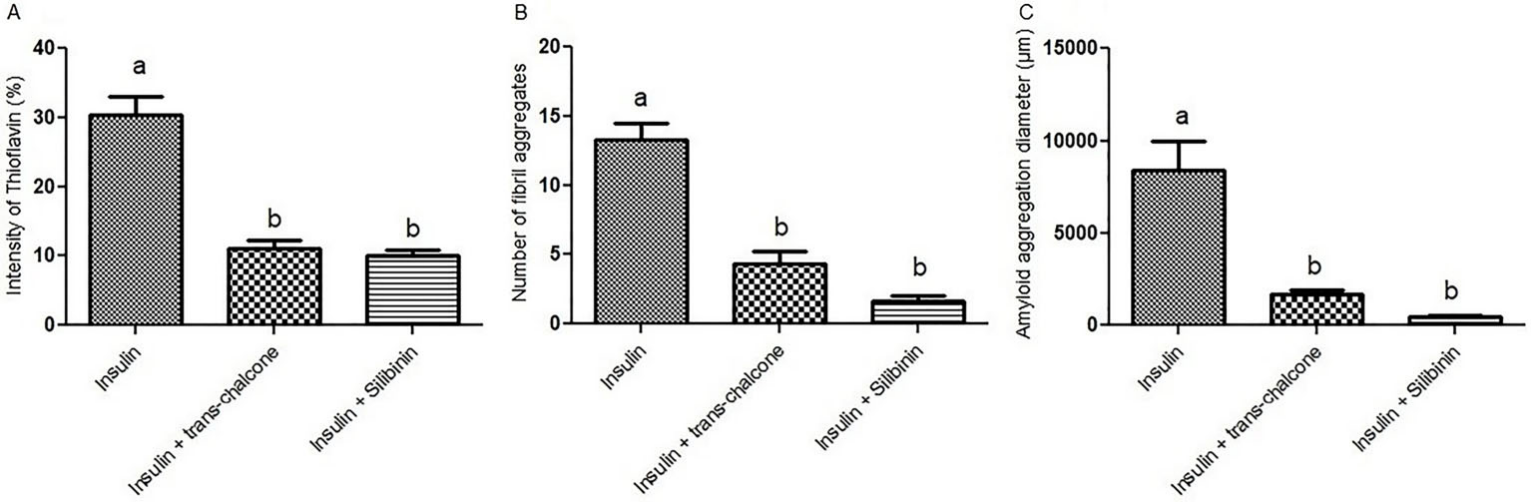
Quantitative analysis of Figure 4 data. Data are reported as the mean and standard error. Different letters indicate statistically significant differences (ANOVA). **A**, Intensity of thioflavin S (%) in the presence and absence of *trans-*chalcone and silibinin, (**B**) number of fibril aggregates in the presence and absence of *trans-*chalcone and silibinin, (**C**) amyloid aggregation diameter (µm) in the presence and absence of *trans*-chalcone and silibinin.

As an additional test to detect amyloid fibril formation, thioflavin S staining, which is amyloid-specific, was also performed. The insulin group showed a significant amount of amyloid deposits (Figure 4A), which was significantly higher than the two other groups (30% stain intensity in Figure 5A). In insulin + *trans-*chalcone and insulin + silibinin groups, staining intensity was about 10% (Figure 4B and C and Figure 5A). The number of fibril aggregates per unit area was also higher in the insulin group, and it seemed that with this rise, the context became more suitable for the development of insulin-amyloid plaques. On the other hand, in insulin + *trans-*chalcone and insulin + silibinin groups, the number of these aggregates was much lower (Figure 4B and C and Figure 5B). It should be noted that aggregate diameters in these two groups were also significantly reduced compared to the those in the insulin group (Figure 5C). The size of aggregate cluster was remarkably greater n the insulin group (reaching about 10,000 µm).

### Passive avoidance test for learning and memory

**Figure 6:**
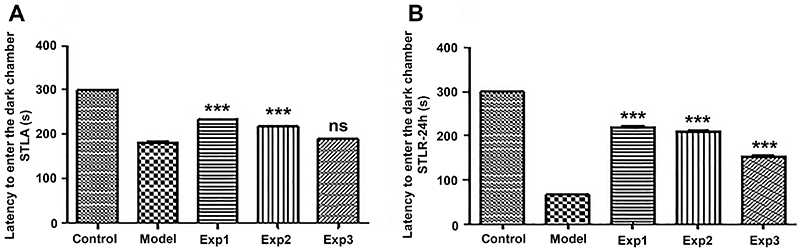
Effect of bilateral microinjection of insulin amyloid fibrils in the presence and absence of *trans-*chalcone. **A**, Step-through latency in the acquisition (STLA) trial in rats of the passive avoidance test. **B**, Step-through latency in the retention (STLR) test after 24 h of the passive avoidance test. Data are reported as means±SE. ***P<0.001 compared with model group (ANOVA). ns: not significant. See Table 1 for group description.

**Figure 7:**
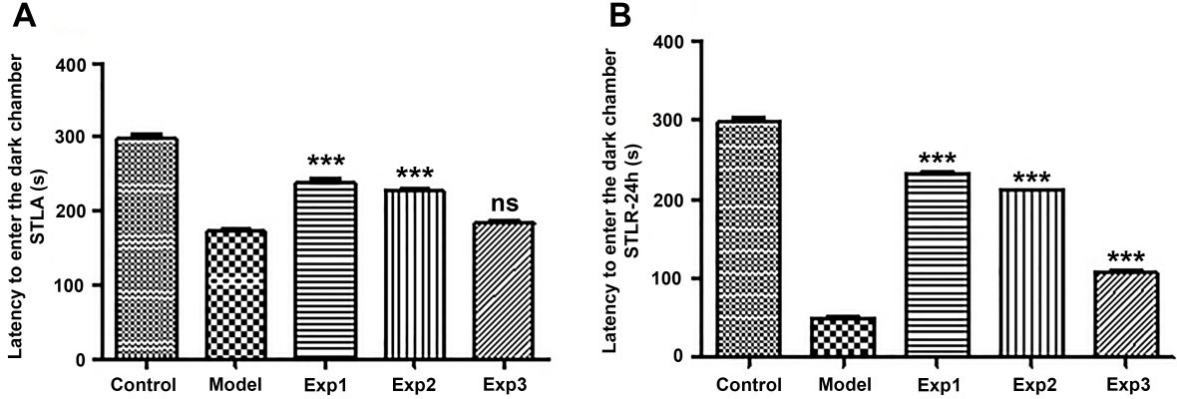
Effect of bilateral microinjection of insulin amyloid fibrils in the presence and absence of silibinin. **A**, Step-through latency in the acquisition (STLA) trial in rats of the passive avoidance test. **B**, Step-through latency in the retention (STLR) test after 24 h of the passive avoidance test. Data are reported as means±SE. ***P<0.001 compared with model group (ANOVA). ns: not significant. See Table 1 for group description.

Findings from the passive avoidance test (shuttle box) on the training day indicated no significant difference between the model group, which received injections of insulin fibrils and phosphate buffer in the hippocampus, and the EXP3 group, which received injections of silibinin or *trans*-chalcone with insulin fibril in the hippocampus. Moreover, statistical analysis showed a significant difference between the model group and EXP1 and EXP2 groups, that is, when compounds were added at the beginning and middle of the fibrillation process. These groups (EXP1 and EXP2) showed better memory than the model group (induced AD group) (P<0.001) (Figures 6A and 7A).

The mean duration of the delay in entering the dark room between the model and experimental groups was evaluated on the day of the test, which was performed 24 h after the training day. Statistical analysis showed a significant difference between the model group and the other treatment groups (P<0.001) (Figures 6B and 7B).

### Histological findings

#### Hematoxylin and eosin (H&E) staining

**Figure 8:**
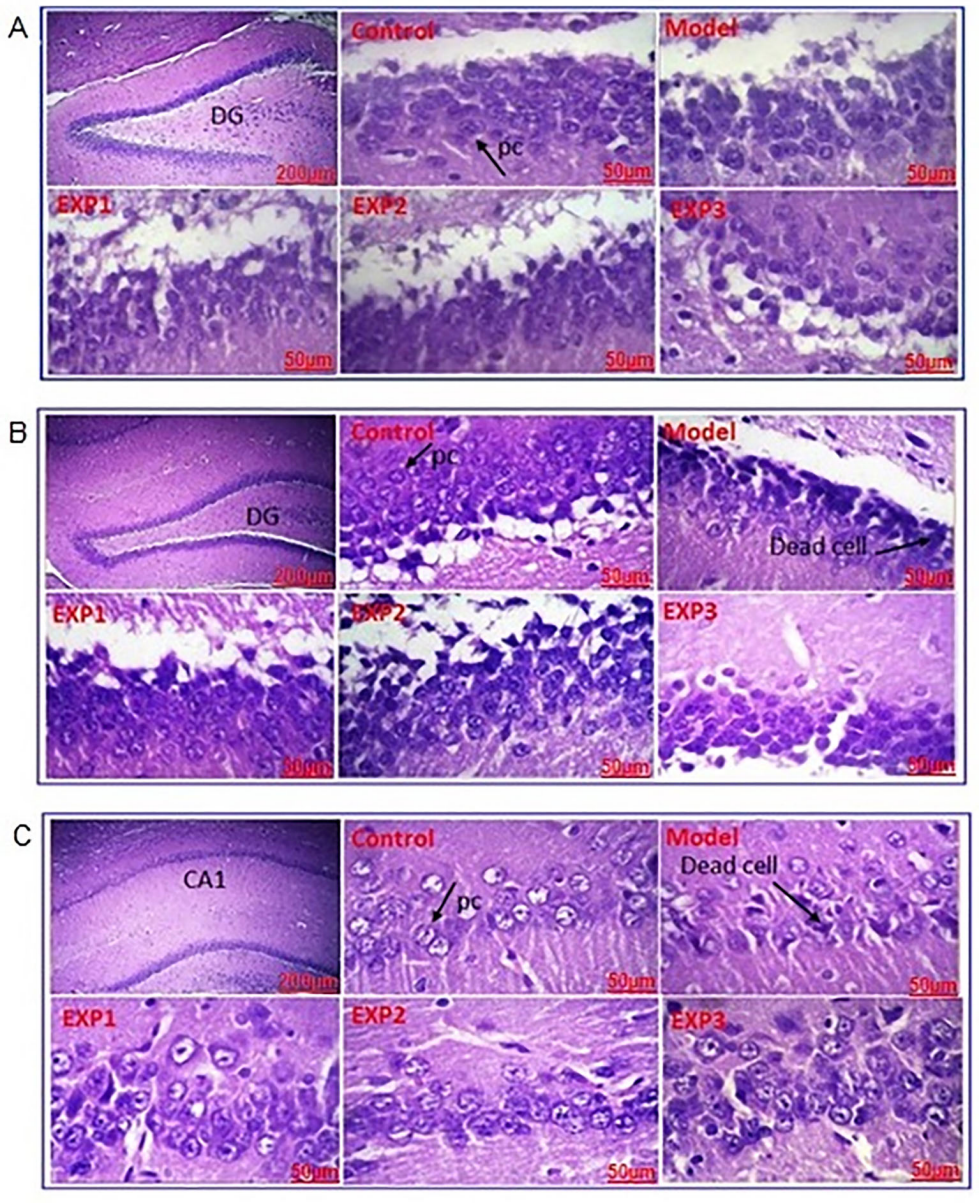
Light microscopic results of hippocampus (dentate gyrus (DG) and cornu ammonis (CA1)) samples stained by hematoxylin and eosin. First image in each box is at ×40 magnification (scale bar 200 μm) and the other images are at ×400 magnification (scale bar 50 μm). Please refer to the Table 1 for group definitions. PC: pyramidal cells. **A**, Insulin amyloid effects in the presence and absence of *trans*-chalcone in the dentate gyrus region, (**B**) presence and absence of silibinin in the dentate gyrus region, and (**C**) presence and absence of silibinin in the CA1 region.

**Figure 9:**
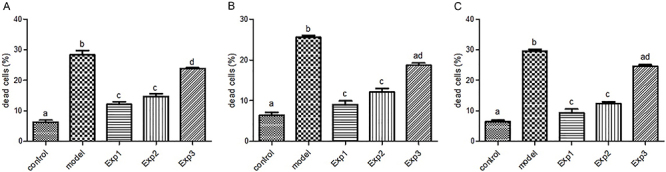
Quantitative analysis of Figure 8 data. Control group represents healthy neurons, model group represents neuronal damage, and treated groups represent mild neuronal injury. In all groups, data were obtained from 5 different animal specimens with 5 random slides and are reported as means±SE. Different letters indicate a statistically significant difference (ANOVA). **A**, Rate of cell death in the presence and absence of *trans-*chalcone in the dentate gyrus region, (**B**) in the presence and absence of silibinin in the dentate gyrus region, and (**C**) in the presence and absence of silibinin in the cornu ammonis region. See Table 1 for group description.

Insulin amyloid effects in the presence and absence of *trans*-chalcone in the DG region are shown in Figure 8A. In the control group, most neurons in the DG were alive, with 5 to 10% of dead neurons, which is significantly lower than in other the groups. On the other hand, in the AD model group, different areas of the hippocampus showed impaired cohesion and structure. Due to tissue destruction, the cell population was reduced in the model group compared to the healthy control group. Overall, the percentage of dead neurons was about 20 to 30%, significantly higher than the control group (Figure 9A).

In the EXP1 and EXP2 groups, the DG tissue was relatively improved and the quality of the neuronal cell population was significantly increased compared to the AD model group.

In the EXP3 group, the number of neurons was still higher compared to the AD model group, but lower than EXP1 and EXP2. When comparing the treatment groups, neurons in the DG region in the EXP1 group appeared to be improved compared to the other two experimental groups. However, the percentage of neurons with cell death in this group was about 10 to 15%, which was not significantly different than the EXP2 group (Figure 9A).

The coronal section of the rat's DG stained with hematoxylin and eosin is shown in Figure 8B. In the control group, the population of neurons in the DG area were mostly alive, and the rate of cell death was lower (6.46±1.21%) than in the model (25.57±0.76%), EXP2 (12.09±1.66%), and EXP3 (18.69±0.93%) groups (P<0.05). In the model group, the tissue showed no clear cohesion and order, and this tissue disruption was more common in the DG than in CA1 (Figure 8). Cell death in the model group (25.57±0.76%) was higher than in the EXP1 (9.06±1.53%), EXP2 (12.09±1.66%), and EXP3 (18.69±0.93%) groups (P<0.05). On the other hand, this region was markedly improved in the EXP1 and EXP2 groups, which were similar [EXP2 (12.09±1.66%) and EXP1 (9.06±1.53%), with no statistical significance], and interestingly, there was no significant difference between the control group and the EXP1 group. In the EXP3 group, the number of neurons was higher compared to the model group, and improvement was observed in this area, although not as much as the other EXP groups [EXP3 (18.69±0.93%) was higher than EXP2 (12.09±1.66%) and EXP1 groups (9.06±1.53%), (P<0.05)] (Figure 9B).

Hippocampus sections of the CA1 region are shown in Figure 8C. Cell death in the control group (6.44±0.973%) was lower than in the model (29.50±1.062%), EXP3 (24.57±0.878%), and EXP2 (12.40± 0.885%) groups (P<0.05), but interestingly, not significantly different from the EXP1 group (9.38±2.05%). Cell death rate was still higher in the model group (29.50±1.06%) than in the other groups: EXP3 (24.57±0.87%), EXP2 (12.40±0.88%), and EXP1 group (9.38±2.05%) (P<0.05). Again, cell death improvement of the EXP3 group was between that of the model group and the other EXP groups (Figure 9C).

Microscopic analysis indicated that there was no change in the DG and CA1 of the hippocampus tissue in the control group, which showed normal histological architecture of Pyramidal cells, while in the AD model group, cell separation due to necrosis and lack of cohesion were observed in these areas. The findings of this study showed that insulin fibrils increased tissue damage, necrosis, and neuronal destruction in DG and CA1 areas. Furthermore, the number of pyramidal cells was reduced, and the number of dead cells was increased in these areas.

In the EXP1 and EXP2 groups, a significant reduction in tissue damage and an increase in tissue cohesion, as well as an increase in the number of pyramidal cells were observed, which indicated the effectiveness of the *trans*-chalcone/silibinin treatment.

In the EXP3 group, tissue damage and necrosis were similar to the model group, and there was no significant effect on damage improvement by insulin fibrils.

#### Thioflavin S staining

**Figure 10:**
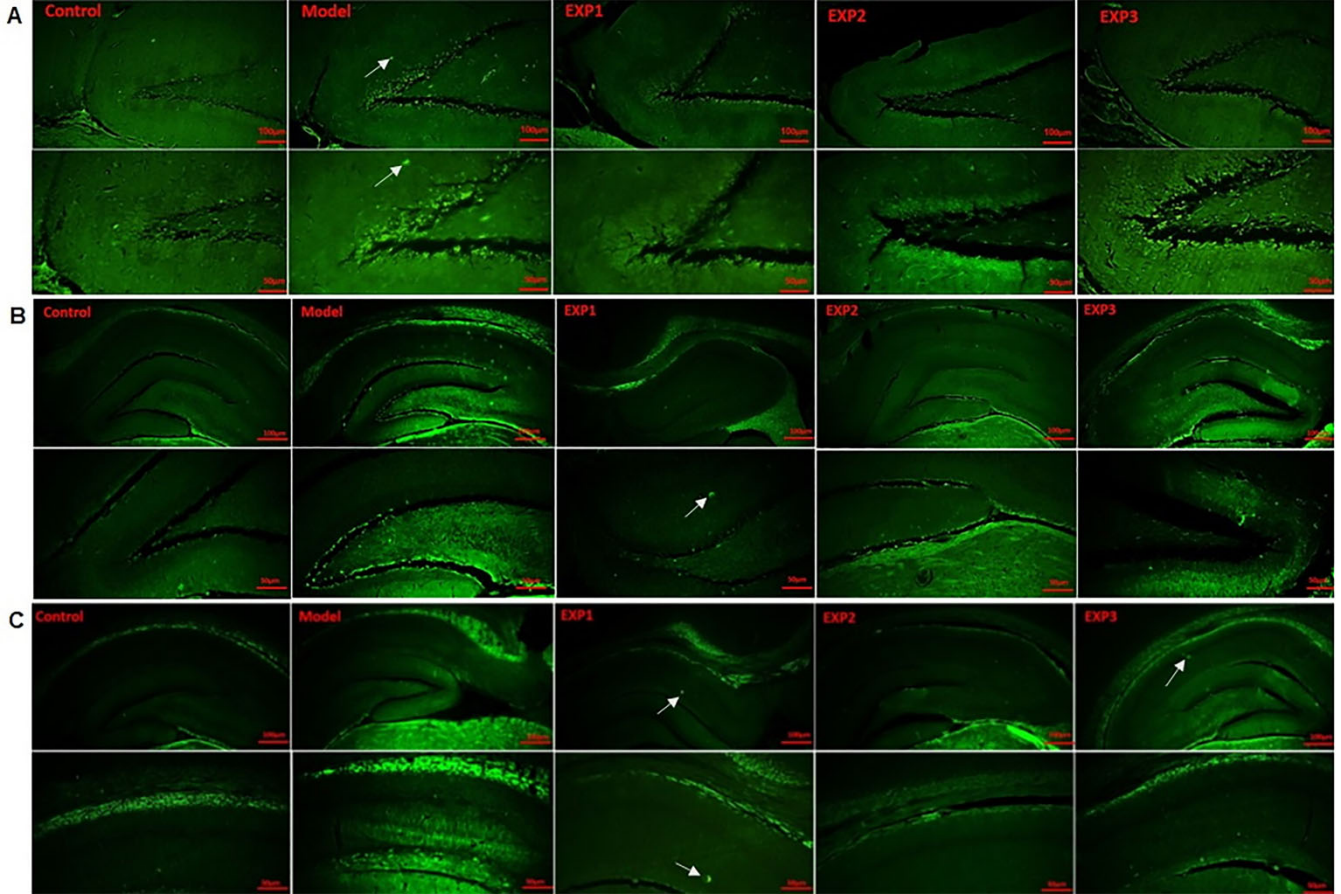
Fluorescence microscopy results of hippocampus samples, showing amyloid fibril deposits highlighted by thioflavin S staining (×40 and ×100, scale bars 100 and 50 μm). Green dots indicate amyloid insulin plaques. White arrows indicate amyloid plaques in the model group. **A**, Insulin amyloid plaques in the presence and absence of *trans-*chalcone in the dentate gyrus region, (**B**) in the presence and absence of silibinin in the dentate gyrus region, and (**C**) in the presence and absence of silibinin in the CA1 region. See Table 1 for group description.

Thioflavin S staining was used on DG slides as a specific probe for amyloid structures. As shown in Figure 10, significant histological changes occurred in the hippocampus upon injection of insulin amyloids.

**Figure 11:**
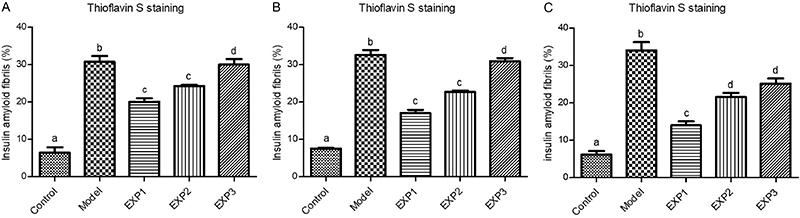
Quantitative analysis of Figure 10 data. Control group had healthy neurons, model group showed neuronal damage, and treated groups had mild neuronal injury. In all groups, data were obtained from 5 different animal specimens with 5 random slides and are reported as means±SE. Different letters indicate statistically significant differences (ANOVA). **A**, Percentage of insulin amyloid fibrils in the presence and absence of *trans-*chalcone in the dentate gyrus region, (**B**) in the presence and absence of silibinin in the dentate gyrus region, and (**C**) in the presence and absence of silibinin in the cornu ammonis region. See Table 1 for group description.

The model group showed a significant increase in plaques compared with control (P<0.001) (Figures 10A and 11A). Compared with the model group, the EXP1 (P<0.001), EXP2 (P<0.001), and EXP3 (P<0.01) groups showed significantly reduced numbers of amyloid plaques in the hippocampus, with the highest reduction in the EXP1 group. The number of plaques in the EXP1 and EXP2 groups was significantly lower than in the EXP3 group (P=0.01) (Figures 10A and 11A).

As shown in Figures 10B and 11B, in the model group and in EXP3, accumulation of plaques (green dots) in the DG was higher compared to the other groups. The percent of plaques in the model group (32.61±6.34%) was also higher than in the EXP3 group (30.91±1.96%) (P<0.05). EXP1 (17.03±1.18%) and EXP2 (22.69±2.59%) groups showed no statistical difference (Figure 11B).

Figures 10C and 11C show amyloid plaque results for the CA1 region of the hippocampus. The observed trend was similar to that of DG: the number plaques in the model group (34±3.915%) was higher than in the EXP1 (13.95±1.915%), EXP2 (21.57±1.92%), and EXP3 (25.10±2.45%) groups (P<0.05), and EXP3 was even higher than the other EXP groups. However, the EXP1 group showed significantly lower levels of plaque compared with EXP3 and EXP2 (P<0.05) (Figure 11C).

### *In silico* test: docking

**Figure 12:**
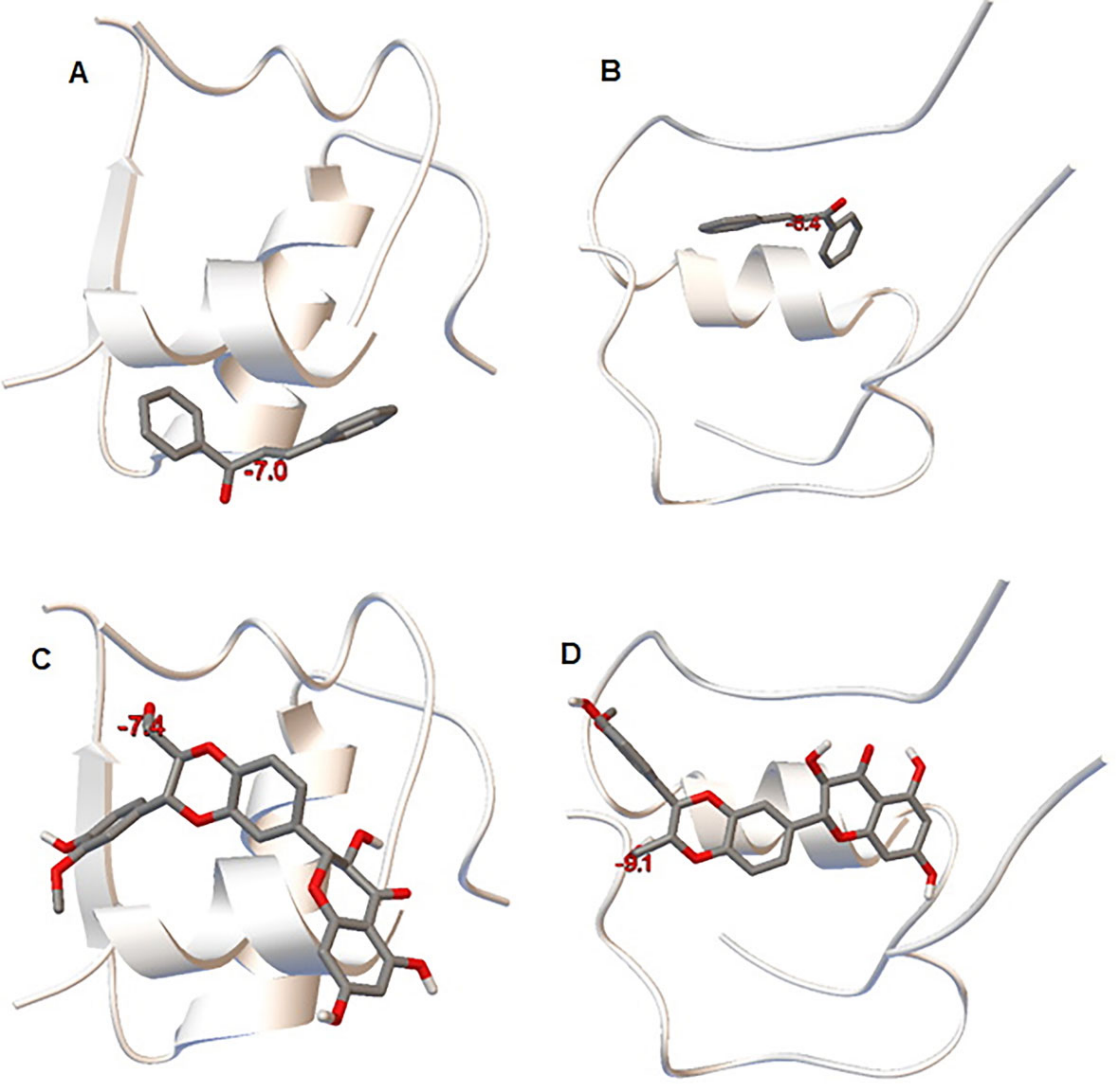
3D representations of *trans-*chalcone interactions with (**A**) native and (**B**) denatured insulin and 3D representations of silibinin interactions with (**C**) native and (**D**) denatured insulin.

The results of silibinin and *trans-*chalcone docking on natural and denatured insulin (amyloid structure) are shown in Figure 12. Both compounds bound to the two forms of the protein, with interaction energy of *trans-*chalcone with the denatured protein being -7.0 and with the natural form, -6.4 kcal/mol, indicating a stronger ligand interaction with the denatured state of the protein. The interaction energy of *trans*-chalcone was weaker compared to silibinin, which had a -9.1 kcal/mol interaction energy with the denatured form and -7.4 kcal/mol with the native form. Silibinin also bound to both protein chains (A and B) in the denatured state, whereas the natural protein only interacted with the insulin A chain (Figure 12). The results indicated the appropriate interaction of silibinin with both forms of the protein.

## Discussion

Animal disease models have limitations in the sense that they can reproduce the disease only to some extent, and as such, results will also be interpreted within that same framework (15). In this study, our AD animal model was generated by intra-hippocampal injection of amyloid fibrils that were formed *in vitro*. This method resulted in plaque formation in the brain of the model animals, as well as memory impairment, which are two hallmarks of AD, and our results focused on those two aspects. One limitation of this model, when used in our time period, could be that in AD patients, AD plaques could be more numerous and extend to other brain regions (16). On the other hand, peptide injections can cause inflammation, which is also a hallmark of AD. However, this method can emphasize the deleterious effects of inflammation, whereas in a more authentic context, inflammation *per se* can have a beneficial side. Injection of a peptide also causes AD symptoms in a relatively short period of time, while in reality, the disease is a slowly developing one, and may involve other processes such as cerebrovascular pathology and other ageing-related manifestations (17). Despite these limitations, peptide injection-based models can be used to assess the anti-amyloid effects of compounds *in vivo*, including in brain tissue, and they provide some hints towards potential lead compounds in this regard.

Our study evaluated the effects of *trans-*chalcone and silibinin on AD model animals and revealed that simultaneous intra-hippocampal injection of *trans-*chalcone or silibinin with insulin fibril was effective in improving the symptoms of induced AD, although both compounds were more effective if present at the time of insulin fibril formation.

Protein assemblies have been extensively studied in recent years in biotechnology and medicine, since they may cause problems in therapeutic protein production and are involved in the development of various diseases (18). The main causes of protein accumulation are very complex and not fully understood, but one of the main strategies proposed as prevention and treatment of protein-related diseases, including neurodegenerative ones, is the use of compounds, usually small molecules that inhibit the fibrillation process. Studies on amyloid aggregations show that all proteins, regardless of being pathogenic or not, should be able to form amyloid fibrils if placed under appropriate conditions. Furthermore, since they all use the same intermolecular mechanisms and interactions in the process of forming amyloid aggregates, fibrils produced by these proteins are structurally similar to the fibrils formed by the Aβ protein in AD. Thus, model proteins can be used for follow-up on amyloid studies. As an example, much research has been carried out on egg white lysozyme, where the inhibitory properties of several aromatic compounds in the formation of lysozyme fibrils have been investigated (19).

AD signs can be induced in rats by driving model proteins such as insulin into amyloid state and injecting them into the rat's brain. Use of regular insulin amyloid fibrils instead of the Aβ peptide is an easier way to obtain an AD model, since insulin is more accessible, available, and cost-effective than the Aβ peptide. In addition to behaving similar to Aβ, model proteins have other advantages such as high stability and high reproducibility (20). From our previous study, we knew that bilateral injection of insulin amyloid fibrils into the hippocampus can generate formation of AD plaques in male rats, which also show reduced memory (13). Our present results in the model group are in good agreement with these studies, both behaviorally and histologically.

Although insulin amyloids are generally considered non-pathogenic, they can be problematic in diabetic patients who regularly inject insulin, especially if they do so repeatedly at the same location. The first case of insulin-induced amyloidosis was presented in 1983; Störkel et al. (21) showed that after 5 weeks of continuous administration, insulin amyloid fibrils were detected.

Weids et al. (22) suggested that stress conditions such as heat lead to protein misfolding and subsequent aggregation. Insulin is the tenth protein and the first extracellular protein found to have a high ability for the creation of amyloid aggregation in humans. In several studies, the formation of insulin amyloid fibrils has been observed under various conditions; for example, at pH 2 and temperature of 57°C (23) or pH 7 and temperature of 37°C (10). If insulin has a hexameric form, a chelator like EDTA can be added to the medium to facilitate the availability of monomeric form at 37°C and pH 7.5, which can then be driven toward amyloid fibril formation (24). The B-chain of insulin plays an important role in amyloid formation, since under certain conditions, the B-chain isolated from bovine insulin has potential to form fibril aggregation. It has also been shown that the C-terminal part of this chain has the highest tendency to form protein aggregates (25).

Herbal medicines have been used for a long time to treat various diseases, including AD. They are now gaining more attention due to their availability and lower cost and are sometimes found to have fewer side effects compared to chemical drugs, to which they are considered a good alternative worldwide (5). Research on inhibiting the formation of amyloid deposits has found that tiny aromatic molecules are very effective in inhibiting inter-protein bindings (26). Some of the inhibitor ligands bind specifically to the natural form of proteins and stabilize proteins against structural changes, while others can directly affect the formation of amyloid fibers by interfering with stacking between beta strands, showing potential therapeutic effect toward misfolding diseases such as AD (27). Accordingly, research into finding new aromatic molecules with inhibitory effects is of considerable interest.

During recent years, particular attention has been paid to *trans-*chalcone, a precursor of flavonoids, because of this simple molecule's wide range of properties. For example, Craggs et al. (28) showed that *trans-*chalcone has neuroprotective effects, especially through antioxidative properties. In another study, we investigated the effect of oral administration of *trans-*chalcone in an AD rat model generated by Aβ injection and found that *trans-*chalcone inhibited amyloid plaque formation in these animals (6).

Silibinin (silybin), a flavonolignans derived from the herbal medicine milk thistle (*Silybum marianum*), is one of the major ingredients of silymarin. In addition to its anti-inflammatory and strong antioxidant features, it is also beneficial in the treatment of cancer, diabetes, and liver disease (7) and has shown anti-amyloidogenic properties (29). Now, silibinin is frequently used to clinically treat cirrhosis, hepatitis, liver poisoning, and fatty liver (7).

This study examined the potential of *trans-*chalcone and silibinin to inhibit the formation of amyloid aggregations of insulin *in vitro.* Besides the fact that the presence of these compounds at the beginning of insulin incubation attenuated the amyloid formation process, adding *trans-*chalcone or silibinin at mid-process of fibril formation still decreased fibril amounts, so the compounds are probably acting at various levels of the aggregation process. Based on the Congo red spectra, silibinin appeared to maintain the protein's native structure while changes in the spectra in the presence of silibinin were much less pronounced compared with *trans-*chalcone (Figure 3). However, at the end of the process, thioflavin S staining *in vitro* did not show a significant difference between the two compounds, although in visual inspection, amyloids that were formed in the presence of silibinin had a more diffuse nature (Figures 4 and 5).

The role of the hippocampus is well known in many memory formation processes. Dentate gyrus is a part of the hippocampus and plays a vital role in learning and memory (30). The results of this study showed that hippocampal injection of fibril insulin caused cognitive impairments associated with learning and memory and reduced CA1 as well as DG neurons. Both compounds have either stimulated neurogenesis in the hippocampus or inhibited the deleterious effects of amyloid structures and improved learning and memory performance of the treated animals, as observed in the behavioral test. Since the pathological nature of insulin fibrils was attenuated when those fibrils were formed in the presence of *trans-*chalcone and silibinin, these compounds may have a potential disintegrating effect on the fibrils. Between the two compounds, silibinin seemed to have a more pronounced effect (Figures 6 and 7).

In histological examination, a lower number of amyloid deposits with markedly smaller diameter were observed upon administration of fibrils formed in the presence of the compounds and, in a less effective manner, when fibrils were injected simultaneously with the compounds (Figures 10 and 11). These results are in good agreement with a study by Urata et al. (9) showing the inhibitory effect of silymarin on fibril formation and subsequently on the number of amyloid plaques formed in the mice brains; this is suggestive of a generalized effect of silibinin on amyloid structure formation. Accordingly, various aromatic compounds have been tested on Aβ amyloid formation and improvement of behavioral tests: Hartmann et al. examined the role of pomegranate juice in reducing the formation of amyloid aggregates (31). Another research has investigated the effect of green tea in reducing amyloid beta aggregation due to the presence of polyphenols (32). Azizi et al. (33) also showed that effective compounds of safflower extract (carvacrol and thymol) also play an effective role in boosting the memory of AD mice. Siahmard et al. (34) have investigated the effect of grape juice. Hosseinzadeh et al. (35) explored the effect of saffron extract, while the effect of water extract of *Elaeagnus angustifolia* was examined by Tamtaji et al. (36). In the same way, Ramshini et al. (37) investigated the direct effect of cinnamon extract and green tea. The results of all these studies indicated that those compounds have the potential to inhibit the formation of amyloid fibrils and improve the memory of rats. This study also agrees with the studies published by Rabiee et al. (38) on the effect of curcumin on amyloid fibrillation of bovine insulin protein. Curcumin exerted its inhibitory effect through interaction with native states, intermediate states, and insulin fibrils. This compound inhibited fibril formation and disrupted pre-formed fibrils. Also, benzofuranone derivatives, which have an aromatic ring possibly located between β-sheet plates, would cause disruption of their aggregation. Yang et al. (39) and Garcia-Alloza et al. (40) in two separate studies reported that curcumin improved spatial memory by inhibiting the formation of amyloid fibrils and had anti-Alzheimer's effects. The overall trend in those studies was an *in vitro* effect of amyloid formation inhibition or disruption of formed aggregates, both of which were observed with our two compounds.

In some of our experiments, silibinin seemed more effective than *trans-*chalcone. The *in silico* results also showed that compared with *trans-*chalcone, silibinin could bind to larger areas of insulin structure, interacting with an important segment of the protein sequence (Figure 12).

It could be suggested that the two compounds are able to inhibit the formation of primary nuclei prone to aggregation by disrupting the interactions between the aromatic amino acids of insulin, thus inhibiting the formation of amyloid fibers. Similarly, they would interact with formed fibrils and disaggregate them to some extent, since the late addition of compounds to fibrils was less successful than adding them at the beginning of the process. In summary, both compounds are interesting with regard to therapeutic potential in amyloid-related diseases and may possess a generic anti-amyloid property.
